# Behavioral Restriction Determines Left Attentional Bias: Preliminary Evidences From COVID-19 Lockdown

**DOI:** 10.3389/fpsyg.2021.650715

**Published:** 2021-04-09

**Authors:** Anna Lardone, Patrizia Turriziani, Pierpaolo Sorrentino, Onofrio Gigliotta, Andrea Chirico, Fabio Lucidi, Laura Mandolesi

**Affiliations:** ^1^Department of Social and Developmental Psychology, Faculty of Medicine and Psychology, Sapienza University of Rome, Rome, Italy; ^2^Department of Psychology, Educational Sciences and Human Movement, University of Palermo, Palermo, Italy; ^3^Institut de Neuroscience des Systemès, Aix-Marseille University, Marseille, France; ^4^Department of Humanities, University of Naples Federico II, Naples, Italy

**Keywords:** executive function, attention, cognition, coronavirus, quarantine, pandemic

## Abstract

During the COVID-19 lockdown, individuals were forced to remain at home, hence severely limiting the interaction within environmental stimuli, reducing the cognitive load placed on spatial competences. The effects of the behavioral restriction on cognition have been little examined. The present study is aimed at analyzing the effects of lockdown on executive function prominently involved in adapting behavior to new environmental demands. We analyze non-verbal fluency abilities, as indirectly providing a measure of cognitive flexibility to react to spatial changes. Sixteen students (mean age 20.75; SD 1.34), evaluated before the start of the lockdown (T1) in a battery of psychological tasks exploring different cognitive domains, have been reassessed during lockdown (T2). The assessment included the modified Five-Point Test (m-FPT) to analyze non-verbal fluency abilities. At T2, the students were also administered the Toronto Alexithymia Scale (TAS-20). The restriction of behaviors following a lockdown determines increased non-verbal fluency, evidenced by the significant increase of the number of new drawings. We found worsened verbal span, while phonemic verbal fluency remained unchanged. Interestingly, we observed a significant tendency to use the left part of each box in the m-FPT correlated with TAS-20 and with the subscales that assess difficulty in describing and identifying feelings. Although our data were collected from a small sample, they evidence that the restriction of behaviors determines a leftward bias, suggesting a greater activation of the right hemisphere, intrinsically connected with the processing of non-verbal information and with the need to manage an emotional situation.

## Introduction

The lockdown imposed to contain the spreading of COVID-19 has made it difficult to maintain healthy habits, such as physical activity and social relations (excluding virtual ones), with consequences on psychological well-being and efficient cognitive functioning. A healthy and active lifestyle determines positive effects on cognition (Hötting and Röder, 2013), improving memory abilities and efficiency of attentional processes and executive-control processes (Colcombe and Kramer, 2003; Grego et al., 2005; Pereira et al., 2007; Winter et al., 2007; Chieffi et al., 2017). Moreover, the continuous interaction with the environment, the practice of physical exercise, and proper nutrition and sleep hygiene, as well as social connectedness, the perception of social support, and other social factors, reduce dysfunctional behaviors, as well as states such as depression and anxiety (Mandolesi et al., 2018; Brooks et al., 2020), improving the quality of life (e.g., Sherbourne and Stewart, 1991; Brown et al., 2012).

The scientific literature concerning similar pandemics, such as the SARS epidemic, demonstrated that quarantine negatively affects psychological well-being, leading to the development of post-traumatic stress symptoms (Reynolds et al., 2008; Castelli et al., 2020; Lardone et al., 2020). To this regard, there are many studies that document a strong relation between COVID-19 quarantine and the onset of stress or stress-related behaviors (Brooks et al., 2020; Lardone et al., 2020; Pisano et al., 2020; Zurlo et al., 2020) among other things, more observed in females (Mazza et al., 2020) and in those who are younger ( ≤ 40 years) (Xiong et al., 2020).

Although to a lesser extent, the effects of quarantine or social isolation on cognitive functioning are mainly documented in terms of behavioral strategies put into action to cope with the restriction period (Boss et al., 2015; for review, see Pera, 2020), recently, it was shown that social isolation determines a worsening of cognitive functioning in later life (Evans, 2019) and in clinical dementia (Tilvis et al., 2000). Accordingly, it is known that loneliness significantly increases the risk of developing dementia (Wilson et al., 2007). While isolation contributes to the acceleration of the aging processes and relates to global cognitive decline in the elderly, in young people, loneliness is correlated to the worsening of executive functions (Cacioppo and Hawkley, 2009), which are strongly affected by the environmental context (Montuori et al., 2019).

Generally, we can define the executive function as the cognitive processes that allow us to select goals; to identify and decide plans of action; to inhibit behaviors; to assume a different behavior in relation to a changing context; to filter interference; to direct, select, and maintain attention on a task; to anticipate the consequences of the actions of others; to reason and solve problems; and to keep the information that is being processed available (Eslinger et al., 1991). In this context, Cacioppo and Hawkley (2009), by means of a dichotic listening task, evidenced that attentional regulation was worse in lonely individuals as compared to non-lonely ones. Another study has evidenced a relation between social isolation and impairment in specific verbal tasks such as verbal fluency and backward digit span (Lara et al., 2019).

Somma and colleagues, analyzing the performances in different peripersonal spatial tasks of a sample of Italian university students before and during the COVID-19 lockdown, showed a significantly leftward bias in the lockdown period. In fact, they observed a tendency to start cancellation from a left-sided item in a cancellation task or to explore first a left-sided arm of a digitized radial arm maze, suggesting more pseudoneglect when behavior is constrained (Somma et al., 2021) and confirming the correlation between social isolation and worsening of the cognitive functioning. The pseudoneglect (Bowers and Heilman, 1980) is a neural pattern of right-hemisphere asymmetry concerning the frontoparietal brain network that plays an important role for orienting and controlling spatial attention (Corbetta et al., 2008; Bartolomeo and Malkinson, 2019). A relative hyperactivity of this attentional right networks might push spatial attention leftward (Gigliotta et al., 2017).

On the basis of the very little available evidence of the effects of the COVID-19 lockdown on cognitive functions in young people, we analyzed the cognitive functioning of a group of young participants before and toward the end of social confinement, in order to assess whether the restriction of behavior had affected their cognitive performance. In particular, we focused on the non-verbal aspects of cognition because during the lockdown period, the individuals were forced to remain at home, hence severely limiting the interaction with all other environmental stimuli, thus reducing the load on spatial competences. For this reason, our attention has focused on non-verbal fluency, which indirectly provides a measure of cognitive flexibility to react to spatial changes. Moreover, during this retest period, we also administered the Toronto Alexithymia Scale (TAS-20, Taylor et al., 1992) in order to detect a possible alexithymia, an affective-cognitive disorder in cognitive processing and emotional regulation (Taylor et al., 1997), often associated with psychopathological conditions (Di Tella and Castelli, 2013; Di Tella et al., 2015) and mainly characterized by difficulty in identifying feelings and in distinguishing between feelings and the bodily sensations of emotional arousal; by difficulty in describing subjective feelings; by restricted imaginative processes, as evidenced by a lack of imagination; and by a stimulus-bound, externally orientated cognitive style (Di Tella and Castelli, 2016).

It is possible to distinguish two types of alexithymia: *primary alexithymia*, a developmental phenomenon thought to be the result of genetic factors, and *secondary alexithymia*, thought to be a consequence of specific conditions as well as stress, chronic disease, or organic processes (e.g., brain trauma or stroke, in this case referring to *organic alexithymia*) (Freyberger, 1977; Spalletta et al., 2001; Messina et al., 2014).

Our hypothesis is that behavioral restriction affects graphic fluency abilities increasing a leftward bias, considering the graphic fluency as spatial competence. To this aim, we used a modified version of the Five-Point Test (m-FPT, Cattelani et al., 2011) that measures the ability of an individual to produce geometric drawings or unique figures within a given time interval, and although it is not a classic test for attention disorders, it allows us to evaluate a shift of the drawings toward the left side of the sheet. Furthermore, recognizing that non-verbal information is mainly processed by the right hemisphere and considering the lockdown period to be an emotionally charged situation, we hypothesize also an alteration of emotional regulation processes, mainly mediated by the right hemisphere, correlated to an increasing secondary alexithymia.

## Materials and Methods

### Participants

Sixteen female psychology and philosophy students from the University of Naples “Federico II” aged between 19 and 24 years (mean age 20.75; SD 1.34), previously evaluated before the start of the COVID-19 lockdown (T1) using a battery of psychological tasks exploring different cognitive domains, have been re-evaluated after roughly 40 days of lockdown (T2) to some of the previously administered tests and to TAS-20 to assess any difficulty in identifying and communicating their feelings and in externally oriented thinking. All tasks administered in both phases (T1 and T2) are reported in Table 1.

**Table 1:** Statistical comparisons between T1 and T2 by means of repeated-measures analyses of variance (ANOVAs) of neuropsychological assessment.

**Cognitive domain**	**Neuropsychological test**	***F* (*df*) value**	***P***	**$\eta_{\text{p}}^{2}$**
Intelligence	Raven's advanced progressive matrices Raven, 1962	*F*_(1, 15)_ = 0.032	*n.s*.	0.002
Verbal working memory	Forward digits Orsini et al., 1987	*F*_(1, 15)_ = 9.57	**0.007**	0.389
	Backward digits Orsini et al., 1987	*F*_(1, 15)_ = 0.27	*n.s*.	0.018
Verbal fluency	Word fluency Carlesimo et al., 1996	*F*_(1, 15)_ = 0.72	*n.s*.	0.046

*The bold values means statistical significant*.

In particular, T1 refers to a period from February 17, 2020, to March 2, 2020, exactly 1 week before the start of the lockdown due to COVID-19 in Italy, while T2 refers to the last 2 weeks of quarantine, specifically from April 16, 2020, to May 2, 2020.

Selection criteria for participant recruitment included normal or corrected-to-normal vision and right-handedness. We included in the sample only students who declared that they did not contract COVID-19 and had no direct or indirect contact with a person affected by the virus. Another eligibility criteria concerned having a printer, with the re-test phase being completely carried out remotely via online meetings on the Microsoft® Teams platform. This condition implied the printing of the protocols. In addition, all participants were in good health and had no history of neurological or psychiatric illness. All students were voluntarily enrolled after written informed consent was obtained. The study was approved by the Local Ethics Committee of the University of Naples “Federico II” (protocol number: 12/2020) and was carried out in accordance with the Declaration of Helsinki. This cohort of students was part of a larger sample to which other tests were also administered, and the results are in the publication phase (Somma et al., 2021).

### Measures

#### Neuropsychological Assessment

In order to evaluate the typical development of all the participants, Raven's Advanced Progressive Matrices (APM; Raven, 1962) were administered in T1 and T2 in digital version through the transposition of matrices on Google Modules. In addition to the assessment of intelligence, verbal working term memory abilities and verbal fluency were evaluated by means of forward and backward digits (Orsini et al., 1987) and word fluency (Carlesimo et al., 1996), respectively.

#### m-FPT

**Figure 1:**
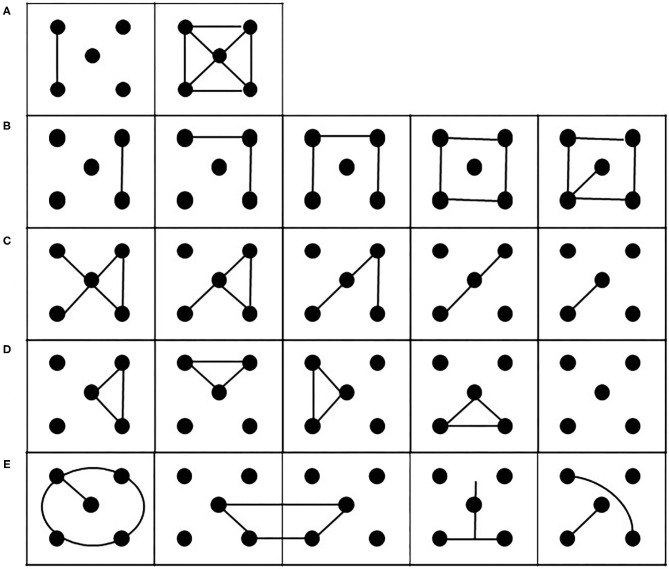
Examples of possible executions of the m-FPT. **(A)** Examples of the two solutions provided by the experimenter to illustrate how to perform the task. **(B)** strategies of addition or **(C)** subtraction elements. **(D)** Strategies of rotation of the patterns produced. **(E)** Examples of possible incorrect patterns.

First developed by Regard et al. (1982), the Five-Point Test (FPT) is a highly reliable non-verbal measure of executive functioning, specifically evaluating the graphic figural fluency (Fernandez et al., 2009). In this study, we have used the m-FTP (Cattelani et al., 2011). In particular, the test measures the ability of an individual to produce geometric drawings or unique figures within a given time interval (3 min). It consists of an A4 sheet with 40 square-shaped matrices each with five dots inside, four of which are arranged at the vertices and one in the middle (Figure 1). The participants are required to connect two or more dots in each square with straight lines. Moreover, they must not repeat twice the same shape and must not draw lines that do not connect dots. This test allows us to analyze three subdomains of executive functions: flexibility, rule breaking, and strategic performance.

The participant is asked: “See these five dots, now with this pencil you have to make several different figures until I say stop. Joining two or multiple dots in each square with a straight or straight line like this (the examiner shows two examples, Figure 1A). But be careful and remember these rules: you can use all the dots or just two, three or four; you don't have to repeat the figures; you must not draw lines that do not connect the dots.”

According to Cattelani et al., 2011, when a student finishes a page, the examiner quickly gives him/her a second sheet of paper while repositioning the first page so that the subject can easily see it.

The parameters evaluated were the following:

Total drawings: number of total drawings made in 3 min;

- Drawings with errors: number of drawings breaking the rules and or repeating previously drawn shapes;
- Error index: number of drawings with errors divided by the number of total drawings multiplied by 100;
- Number of unique drawings: calculated by subtracting the number of drawings with errors from the number of total drawings;
- Strategy index: number of drawings with strategy divided by number of unique drawings;
- Percentage of dots considered on the right: the number of dots on the right forming the drawing divided by the number of total dots multiplied by 100;
- Percentage of dots considered on the left: number of dots on the left forming the drawing divided by the number of total dots multiplied by 100; and
- Percentage of dots considered on the center: number of dots considered on the middle left forming the drawing divided by the number of total dots multiplied by 100.

#### TAS-20

The TAS (Taylor et al., 1992) and a subsequent 20-item form (TAS-20; Bagby et al., 1990) used in this study are the most used and most reliable self-assessment questionnaire for measuring alexithymia, an affective-cognitive disorder characterized by difficulty in identifying and describing owns emotions and in being interested in understanding those of others (Nemiah and Sifneos, 1970). The TAS-20 is made of three subscales, each capturing one of these aspects of the construct of alexithymia (Bressi et al., 1996): the *Difficulty Describing Feelings subscale* (DDF) consisted of five items (2, 4, 11, 12, and 17), the *Difficulty Identifying Feeling subscale* (DIF) consisted of seven items (1, 3, 6, 7, 9, 13, and 14), and the *Externally Oriented Thinking subscale* (EOT) measuring the tendency of individuals to focus their attention externally consisted of eight items (5, 8, 10, 15, 16, 18, 19, and 20).

In TAS-20, the subjects respond through a 5-point Likert scale, whereby 1 indicates strongly disagree and 5 indicates strongly agree. Items 4, 5, 10, 18, and 19 are negatively keyed. The total alexithymia score is the sum of the responses to all 20 items, while the score for each subscale factor is the sum of the responses to that subscale. Subjects with a score equal to or >61 are considered alexithymic, and those with a score equal to or <50 are considered non-alexithymic, with a borderline area between 50 and 60.

### Procedures

In phase T1 (1 week before the start of the COVID-19 lockdown), the participants have been tested in a quiet room of the University of Naples Federico II. In the room, there was a table with chairs, and the participant sat in front of the experimenter. Each participant was first administered the neuropsychological tasks and then the m-FPT, for a total of about 30 min.

In relation to social distancing measures, the second retest phase (T2, during the last 2 weeks of the quarantine) took place completely remotely via online meetings on the Microsoft® Teams platform, a unified communication and collaboration platform that combines chat, teleconferencing, content sharing, and application integration. This way, it was easy to administer all the tests used in T1, as well as the TAS-20 and the m-FPT. In fact, each student was provided with the test file in pdf, which was printed before the call by the experimenter. This way, the test took place under the full supervision of the experimenter. At the end of the test, the student took a picture of the page/pages with the drawings and sent it/them to the experimenter.

### Statistical Analysis

All statistical analyses were conducted with SPSS Statistics 22 software. For each comparison of T1 vs. T2, we used a repeated-measures analyses of variance (ANOVA). *P* values <0.05 were considered statistically significant.

Some data were tested by means of two-tailed Spearman's correlation analysis between the results of the TAS-20 and the increment of dots touched on the left at T2 in m-FPT and the worsening of forward digits, considering for both the Δ as the difference between T2 and T1.

## Results

The results are reported as *F* statistic (*F*), statistical significance (*P*), and bias effect size estimation ($\eta_{\text{p}}^{2}$).

### Neuropsychological Tests

The statistical comparisons of the results obtained when comparing T1 vs. T2 on the previously described neuropsychological tests are shown in Table 1. We found a significant difference only for the forward digits test in which the participants exhibit a worsening in T2 (T1 = 6.5 ± 0.63; T2 = 5.9 ± 0.57).

### m-FPT

**Figure 2:**
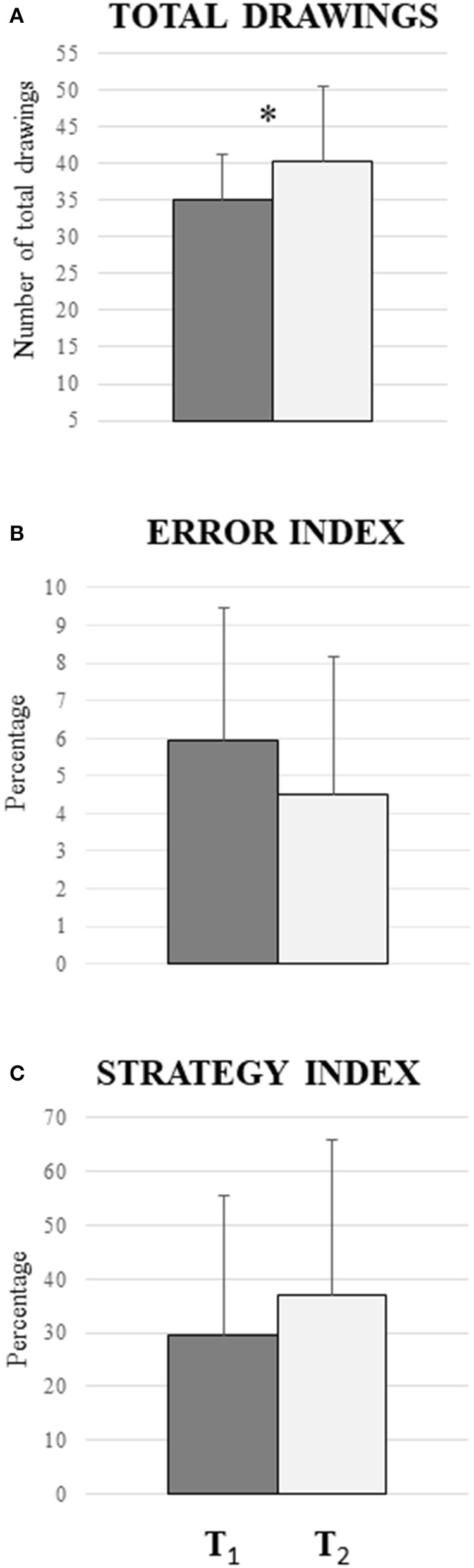
Comparison between the periods before the COVID-19 lockdown (T1) and during the COVID-19 lockdown (T2) in m-FPT. **(A)** Significant difference in total drawings parameter (*P* = 0.024): the participants produced more drawings during the quarantine. **(B)** No significant difference in the error index (*P* = 0.33), indicating that the percentage between the violations of the rule and the repeated drawings does not vary between the two phases. **(C)** No significant differences in the strategy index (*P* = 0.78): the result indicates that there are no changes in the increase of drawings with strategies. Vertical bars indicate SD.

The comparison between T1 and T2 revealed a significant difference between T1 and T2 in the total drawings parameter [*F*_(1, 15)_ = 6.27; *P*: 0.024; $\eta_{\text{p}}^{2}$ = 0.295) as shown in Figure 2A]. A different pattern was observed in the comparison between T1 and T2 in relation to drawings with resulting errors similar to those in T1 and T2 [*F*_(1, 15)_ = 0.24; *P*: 0.63; $\eta_{\text{p}}^{2}$ = 0.016]. This result is also reflected in the error index [*F*_(1, 15)_ = 1.0; *P*: 0.33; $\eta_{\text{p}}^{2}$ = 0.016; Figure 2B] and in the number of unique drawings [F_(1, 15)_ = 1.36; *P*: 0.78; $\eta_{\text{p}}^{2}$ = 0.019], which are not significantly different between T1 and T2. Also, in the strategy index, there are no significant differences between T1 and T2 [F_(1, 15)_ = 1.01; *P*: 0.33; $\eta_{\text{p}}^{2}$ = 0.063, Figure 2C].

**Figure 3:**
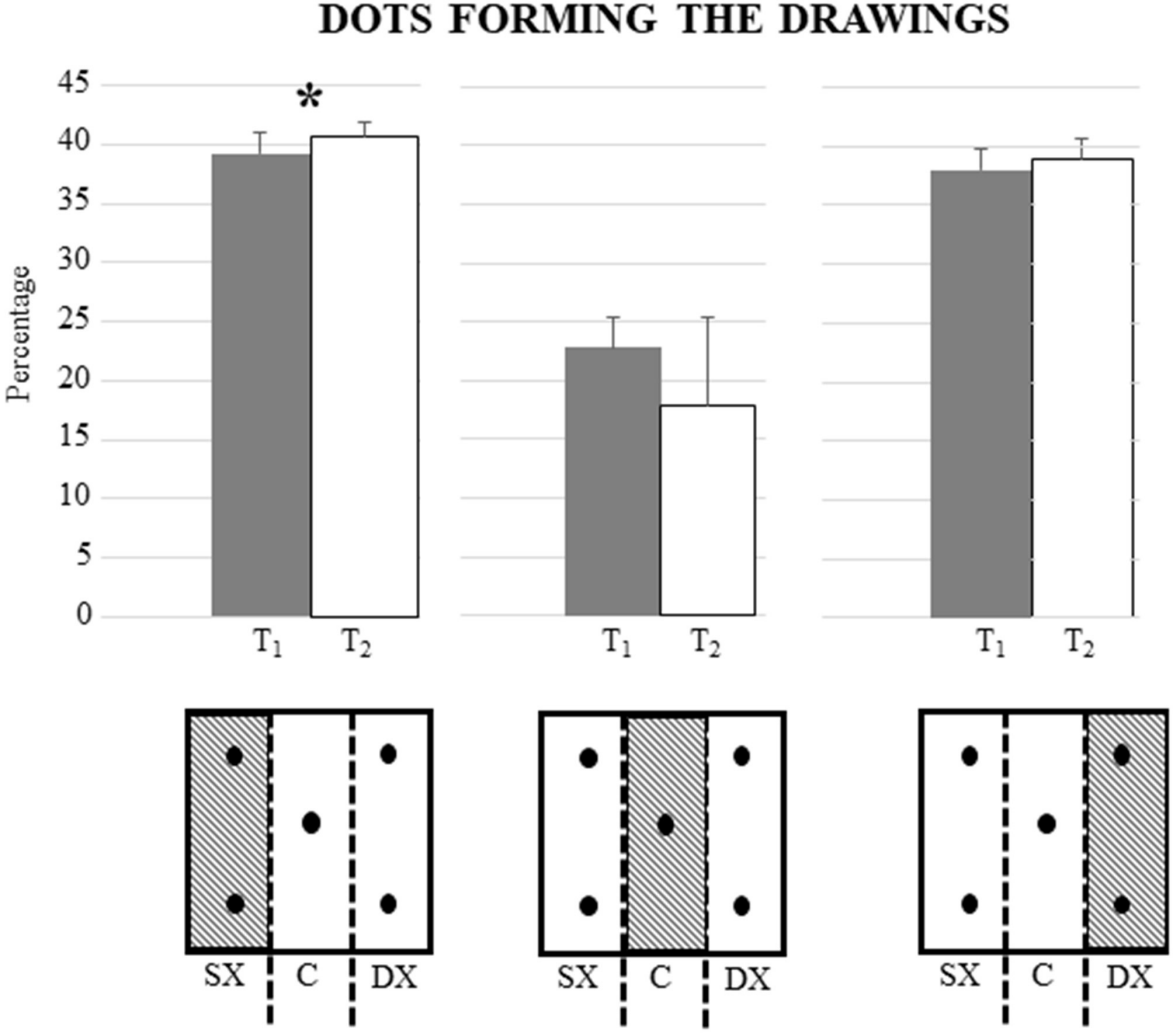
Differences in mean percentage of dots on the left, right, and middle of the boxes between the period before the COVID-19 lockdown (T1) and during the COVID-19 lockdown (T2). There is a significant difference only for the percentage of dots on the left the boxes forming the drawings (*P* = 0.01). Vertical bars indicate SD. In the lower part of the figure, there is a schematic representation of the division of the box into the left, middle, and right.

Interestingly, as shown in Figure 3, when the percentage of dots forming the drawings (located either on the right or left or on the middle of each box in both phases) was considered, significant differences were found only for the percentage of dots on the left [*F*_(1, 15)_ = 7.87; *P*: 0.01; $\eta_{\text{p}}^{2}$ = 0.34]. The percentage of dots on the right, on the other hand, was not significantly different [F_(1, 15)_ = 2.04; *P*: 0.17; $\eta_{\text{p}}^{2}$ = 0.12]. Also, the percentage of dots on the middle remained unchanged [F_(1, 15)_ = 0.03; *P*: 0.96; $\eta_{\text{p}}^{2}$ = 0.00].

#### TAS-20

Among our 16 participants, only four were found to be alexithymic with an average score of 65. The rest of the group (12 students) obtained an average score of 53. Eight of these 12 students obtained an overall score that falls in the critical range of 50–60.

One-way ANOVA revealed a significant difference among three subscales (DDF, DIF, and EOT) [*F*_(2, 45)_ = 18.44; *P*: 0.000]. *Post hoc* comparisons revealed that each subscale is different (DDF vs. DIF: *P* = 0.04; DDF vs. EOT: *P* = 0.000; DIF vs. EOT: *P* = 0.03).

**Figure 4:**
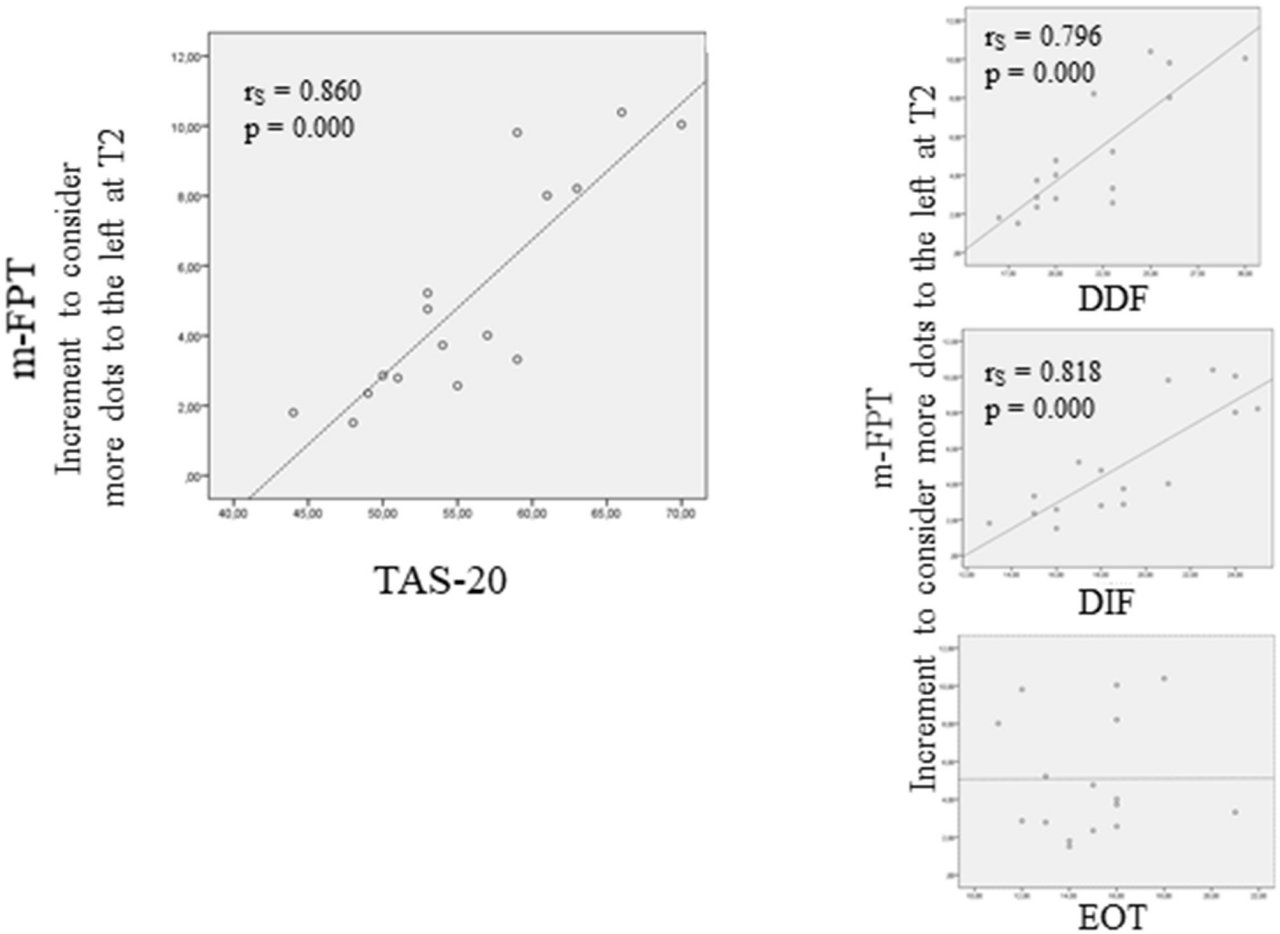
Correlation (Spearman rho) between and the tendency of the m-FPT to shift to the left side at T2 and the TAS-20. On the left side is shown the correlation between the difficulty to identify and describe feelings, as well as externally oriented thinking, and the increment of dots touched on the left at T2. On the right side of the figure is shown the correlations between the different TAS-20 subscales and bias to the left in T2.

To assess a potential relationship between the difficulty to identify and describe feelings, as well as externally oriented thinking, and the tendency of the m-FPT to shift to the left side at T2, a two-tailed Spearman correlation analysis was performed between the results of the TAS-20 and the increment of dots touched on the left at T2. Results showed a significant positive correlation between the bias to left and the TAS-20 (*r*_S_ = 0.860, *P* = 0.000) (Figure 4). Then, correlations between the different TAS-20 subscales and bias to left in T2 were also conducted. Results showed significant correlations between the bias to the left and the DDF (*r*_S_ = 0.796, *P* = 0.000) and the DIF (*r*_S_ = 0.818, *P* = 0.000), while no significant correlation resulted when considering the EOT (*r*_S_ = 0.144, *P* = 0.565).

#### Analysis of Correlation With Neuropsychological Scores

To assess a potential relationship between the digits forward span and the left bias in m-FPT at T2, a two-tailed Spearman correlation analysis was performed. No significant correlation was evident (*r*_S_ = −0.157, *P* = 0.561).

To assess a potential relationship between the difficulty to identify and describe feeling and to think externally and the worsening in span at T2, a two-tailed Spearman correlation analysis between the results of the TAS-20 and the worsening in span at T2 was performed. The results failed to show any significant correlation (*r*_S_ = −0.161, *P* = 0.553). Similar results rose when the correlations were made between the different TAS-20 subscales and the worsening in span at T2 (DDF: *r*_S_ = −0.110, *P* = 0.684; DIF: *r*_S_ = −0.054, *P* = 0.440; EOT: *r*_S_ = −0.208, *P* = 0.565) (Figure 4).

## Discussion

In the present study, we had the great opportunity to test executive functions in a group of students before and during the lockdown imposed by the Italian government, after roughly 40 days of enforced lockdown. This allowed us to compare their performances before (T1) and during the period of enforced behavioral restrictions (T2). In particular, we focused on the effects of the COVID-19 lockdown on non-verbal fluency abilities, which indirectly provide a measure of flexibility of the individual to react to spatial and environmental changes. To study this cognitive domain, we used the m-FPT, which allows us to detect a non-verbal measure of executive functioning and evaluates with a high degree of reliability the graphic figural fluency in a peripersonal space.

We observed that the period of behavioral restrictions determines an increase in spatial fluency ability. In fact, in T2, we see more drawings produced as compared to T1 (Figure 2A). This significant trend does not reflect itself in the other parameters evaluated, such as the error and strategy index (Figures 2B,C). A possible explanation could be that our sample is composed of typical-development students without cognitive deficits, as demonstrated by the normal values of the Raven matrices. Moreover, the overlapping scores of the Raven matrices in T1 and T2 suggest that the effects of behavioral restriction do not reflect a change in global cognitive functioning but, rather, concern specific cognitive domains. The worsening of the forward digits in T2 (Table 1) suggests that the verbal working memory could be specifically affected by the lockdown. A speculative remark might be based on the evidence that the working memory is strongly correlated to noradrenergic activity (Robertson, 2014), and living in a restricted environment, such as during a lockdown, might reduce activation and alertness and hence the noradrenergic tone, which in turn induces a worsening in the working memory performance. Unfortunately, since the second part of the test (T2) was carried out completely at a distance, we could not add the Corsi test (Spinnler and Tognoni, 1987) to the assessment of the spatial working memory, and hence, we could not investigate whether the worsening is specific for verbal working memory or also concerns the spatial domain.

However, the lockdown does not seem to have an effect on verbal fluency. The difference between the verbal domain (i.e., between verbal working memory which worsens during lockdown and verbal fluency which remains stable) could be explained by the fact that our participants are students and therefore continuously training verbal working memory by reading and repeating. Therefore, verbal fluency abilities continue to be exercised despite the lockdown imposing behavioral restrictions. In accordance with this hypothesis, several clinical evidences demonstrate that cognitive exercise increases the cognitive reserve (Stern, 2002; Mandolesi et al., 2017; Gelfo et al., 2018; Serra and Gelfo, 2019). In addition, it is to notice a worsening in forward digits test in T2 explainable for example with the fact that the social isolation determines an impairment in specific verbal tasks such as verbal fluency and backward digit span (Lara et al., 2019). This suggestion is also in accord with the “hemispheric activation model” (Kinsbourne, 1970; Bowers and Heilman, 1980), proposing that the distribution of attention in space is biased contralaterally to the more activated hemisphere. We can speculate that spatial processing activated the right hemisphere more strongly than the left language-dominant hemisphere. This resulted in attentional shifting attention toward the left hemispace.

Interestingly, in the m-FPT, there is a shift to the left side during the lockdown as compared to habitual living conditions (T1) (Figure 3). In fact, analyzing the percentage of dots touched on the right or left of each box of m-FPT, we observed an increment of the dots on the left of each box, suggesting thus an increment of pseudoneglect. These data are in agreement with a previous study conducted in the same experimental condition in which periods before and during COVID-19 lockdown are compared (Somma et al., 2021), which evidenced increased selective spatial attention to the left during lockdown in multiple peripersonal visuospatial tasks, such as cancellation task and a digitized version of the table radial arm maze task (Foti et al., 2020). The authors hypothesized that the stressful conditions experienced by the participants during the quarantine, as measured by the COVID-19 Student Stress Questionnaire (CSSQ; Zurlo et al., 2020), might have increased the activity of the right hemisphere attention networks (Somma et al., 2021), thus causing the observed leftward bias. Even more recently, in support of the hypothesis of greater activation of the right hemisphere in specific environmental conditions, Spreng et al. (2020) have observed consistent volumetric alterations in right inferior parietal and cingulo-opercular regions as well as in the dorsolateral prefrontal cortex in lonely individuals, thus supporting a neural model of loneliness. This evidence is in accordance with the hypothesis of an attentional right network driving spatial attention leftward (Gigliotta et al., 2017).

The greater involvement of the right hemisphere in conditions of behavioral restriction is compatible with the significant positive correlation between the tendency of the m-FPT to shift the attention to the left side and the score obtained by the TAS-20, which allows us to detect difficulties in identifying and describing feelings and in externally oriented thinking. Furthermore, by breaking down the TAS-20 into its three subscales and correlating them to the left increment in the m-FPT, we obtained positive correlations only for the difficulty to describe feelings and the difficulty to identify feelings subscales. These data suggest that the behavioral restriction determines a difficulty in the description and identification of feelings, which are mainly related to the cerebral networks of the right hemisphere. Constantly putting into action barrier gestures, such as disinfecting hands, wearing masks, and maintaining social distancing, might prevent people from fully focusing on the processing of feelings. Instead, the lack of correlation between the left increment in the m-FPT and the externally oriented thinking subscale could indicate that, even during the period of social restriction, one is oriented toward the outside and toward others precisely because humans are “ultra-social animals” (Tomasello, 2014).

In support of the idea that the spatial attentional bias is modulated by emotional states, we recall that patients with Parkinson's and individuals with post-traumatic stress disorder have pathological values at TAS-20, in fact being alexithymic (Salazar et al., 2019).

### Limitations and Future Research Perspectives

The strength is that this study is one of the few that investigated the effects of behavioral restriction in young people during the COVID-19 lockdown. However, there are at least three main limitations, which cannot be overcome but which warrant caution when interpreting the data.

Firstly, the low number of participants does not allow us to generalize completely the results. However, considering that the previous study by Somma et al. (2021) has analyzed a sample of almost 100 female students and found an increasing spatial pseudoneglect, we feel that a behavioral restriction of even a few days causes a leftward spatial attentional bias. Connected to this point, it should be noticed that m-FPT was not developed to study attentional disorders. However, the parameters we have added allow us to evaluate the shift in spatial attention. Another weakness concerns the limited cognitive evaluation made in the present study. We could not analyze all the cognitive domains because these subjects were tested in T1 for other purposes. With the COVID-19 lockdown, we continued to monitor these students to retest them (T2) with a similar modality. This meant that we could not use all the battery proposed in T1 also in T2.

The greatest drawback is that TAS-20 was administered only in T2. Not having the evaluation of the participants in T1, we cannot be sure in characterizing the emotional disorder as secondary alexithymia, although there are several evidences suggesting that relevant environmental factors and significative events occurring during the life determine secondary alexithymia (Messina et al., 2014).

In addition, the administration of TAS-20 only at T2 evidences a methodological issue. Surely, in a normal situation, rigor in research would have prevailed. However, being in a particular historical moment, we believe that any data and evidence must be documented and told.

The present data allow us to document that behavioral restrictions, even for a limited period of time, affects the processing of spatial information in terms of a left attention bias, allowing us to reflect on future intervention and support programs for other lockdowns, in which it will be necessary to study in depth and completely the effects on verbal and non-verbal cognitive functioning just as it will be more appropriate to consider the psychological effects that fall on emotional processing.

Future research must be directed toward the development of protocols capable of analyzing the effects of any lockdowns on all segments of the population, taking into account the different, and interrelated aspects that contribute to cognitive functioning, and must be directed toward the implementation of psychological support programs to avoid the onset of mental illness.

## Data Availability Statement

The raw data supporting the conclusions of this article will be made available by the authors, without undue reservation.

## Ethics Statement

The studies involving human participants were reviewed and approved by Local Ethics Committee of the University of Naples Federico II [Protocol number: 12/2020]. The participants provided their written informed consent to participate in this study.

## Author Contributions

AL, PS, AC, and OG tested participants. AL, PT, FL, and LM wrote the paper. All authors read, revised, and approved the final manuscript, designed research, and analyzed data and discussed data.

## Conflict of Interest

The authors declare that the research was conducted in the absence of any commercial or financial relationships that could be construed as a potential conflict of interest.
